# Microbial diversity and community structure across environmental gradients in Bransfield Strait, Western Antarctic Peninsula

**DOI:** 10.3389/fmicb.2014.00647

**Published:** 2014-12-16

**Authors:** Camila N. Signori, François Thomas, Alex Enrich-Prast, Ricardo C. G. Pollery, Stefan M. Sievert

**Affiliations:** ^1^Instituto de Microbiologia Professor Paulo de Góes, Universidade Federal do Rio de JaneiroRio de Janeiro, Brazil; ^2^Laboratório de Biogeoquímica, Instituto de Biologia, Universidade Federal do Rio de Janeiro, Centro de Ciências da SaúdeRio de Janeiro, Brazil; ^3^Biology Department, Woods Hole Oceanographic InstitutionWoods Hole, MA, USA; ^4^Department of Environmental Change, Linköping UniversityLinköping, Sweden

**Keywords:** Antarctica, pyrosequencing, microbial community structure, environmental factors, microbial oceanography, climate change

## Abstract

The Southern Ocean is currently subject to intense investigations, mainly related to its importance for global biogeochemical cycles and its alarming rate of warming in response to climate change. Microbes play an essential role in the functioning of this ecosystem and are the main drivers of the biogeochemical cycling of elements. Yet, the diversity and abundance of microorganisms in this system remain poorly studied, in particular with regards to changes along environmental gradients. Here, we used amplicon sequencing of 16S rRNA gene tags using primers covering both Bacteria and Archaea to assess the composition and diversity of the microbial communities from four sampling depths (surface, the maximum and minimum of the oxygen concentration, and near the seafloor) at 10 oceanographic stations located in Bransfield Strait [northwest of the Antarctic Peninsula (AP)] and near the sea ice edge (north of the AP). Samples collected near the seafloor and at the oxygen minimum exhibited a higher diversity than those from the surface and oxygen maximum for both bacterial and archaeal communities. The main taxonomic groups identified below 100 m were Thaumarchaeota, Euryarchaeota and Proteobacteria (Gamma-, Delta-, Beta-, and Alphaproteobacteria), whereas in the mixed layer above 100 m Bacteroidetes and Proteobacteria (mainly Alpha- and Gammaproteobacteria) were found to be dominant. A combination of environmental factors seems to influence the microbial community composition. Our results help to understand how the dynamic seascape of the Southern Ocean shapes the microbial community composition and set a baseline for upcoming studies to evaluate the response of this ecosystem to future changes.

## Introduction

The Southern Ocean is currently subject to intense investigations, mainly related to its importance for global biogeochemical cycles and its alarming rate of warming in response to climate change (e.g., Smetacek and Nicol, 2005; Schofield et al., 2010; Luria et al., 2014). This area of the world's ocean accounts for a third of the global ocean CO_2_ uptake, and thus plays a major role in the Earth's carbon cycle. The Southern Ocean is a large and heterogeneous biogeochemical system, comprising both highly productive coastal and ice-edge waters that contrast with open ocean waters where primary production is to a large extent limited by the availability of the micronutrient iron. In particular, the Western Antarctic Peninsula (WAP) is considered as one of the main areas experiencing rapid regional warming (Anisimov et al., 2007), and is the only one with a maritime climate—making it an ideal place to monitor, understand and predict the impacts of climate change on marine ecosystems (Schofield et al., 2010).

Marine microorganisms play a critical role in ecosystem functioning and are the main drivers of the biogeochemical cycling of elements. They dominate in abundance, and diversity, and are responsible for the majority of the metabolic activity of the ocean (Azam and Malfatti, 2007). The processing of organic matter by Bacteria and Archaea is considered a major carbon-flow pathway, and its variability can change the overall pattern of carbon flux (Azam, 1998). Yet, the diversity, activity, and abundance of microorganisms in the Southern Ocean remains poorly studied, in particular with regards to changes along environmental gradients (e.g., Wilkins et al., 2013a,b; Luria et al., 2014). The need of studying the microbial diversity in conjunction with physical and chemical observations (considering both temporal and spatial scales) to understand the role of the microbes in oceanic ecosystems has been previously noted (Karl, 2007). Yet, it is only through recent advances in sequencing technology that it has become feasible to perform these kinds of studies at the required scale and resolution (DeLong and Karl, 2005; Sogin et al., 2006; Frias-Lopez et al., 2008; Yooseph et al., 2010). Some more recent studies have shown that oceanographic factors such as the physical-chemical and biological properties of the water masses (Agogué et al., 2011), advection of cells by water currents (Wilkins et al., 2013a), and seasonal evolution of the upper water column (Luria et al., 2014) have a role in shaping the microbial community structure in the Southern Ocean.

The Southern Ocean is characterized by a complex interplay of environmental variables that may influence the composition of the microbial communities, and at present, studies are still at the stage of establishing reference values for assessing the temporal and spatial dynamics of the microbial communities in the water column (e.g., Wilkins et al., 2013b; Luria et al., 2014). In a recent study, Luria et al. (2014) studied the microbial communities at 4 stations and 2 depths (10 and 100 m) on the continental shelf of the WAP, providing a reference point for future investigations. In the present study, we assessed the composition and structure of bacterial and archaeal communities at 10 stations in a region to the north of that studied by Luria et al. (2014), collecting samples from 4 different depths (from surface to seafloor) influenced by contrasted water masses. Combined with the measurement of various environmental parameters, our study contributes to an increased understanding of the factors that drive the diversity of microbes in this dynamic and rapidly changing region of the Southern Ocean, which is paramount to predict responses of this important biome to future changes.

## Materials and methods

### The study area

The Bransfield Strait (BS) is influenced by different water masses. In this area, cold and salty waters enter from the Weddell Sea (called Transitional Zonal Water with Weddell Sea influence, TWW), while water from the Bellingshausen Sea forms an overlaying well-stratified relatively warm and fresh surface layer (called Transitional Zonal Water with Bellingshausen influence, TBW) (Tokarczyk, 1987; Garcia et al., 1994; Sangrà et al., 2011). BS is also an area with a high biological productivity, all the way from phytoplankton and zooplankton to whales (Zhou et al., 2002; Dalla Rosa et al., 2008). The dynamic hydrographic structure is also reflected in the chlorophyll distribution, as seen by Figueiras et al. (1998), with concentrations lower than 0.5 mg.m^−3^ in surface Weddell Sea waters and higher than 1 mg.m^−3^ in surface waters with Bellingshausen Sea influence.

### Sampling strategy

**Figure 1:**
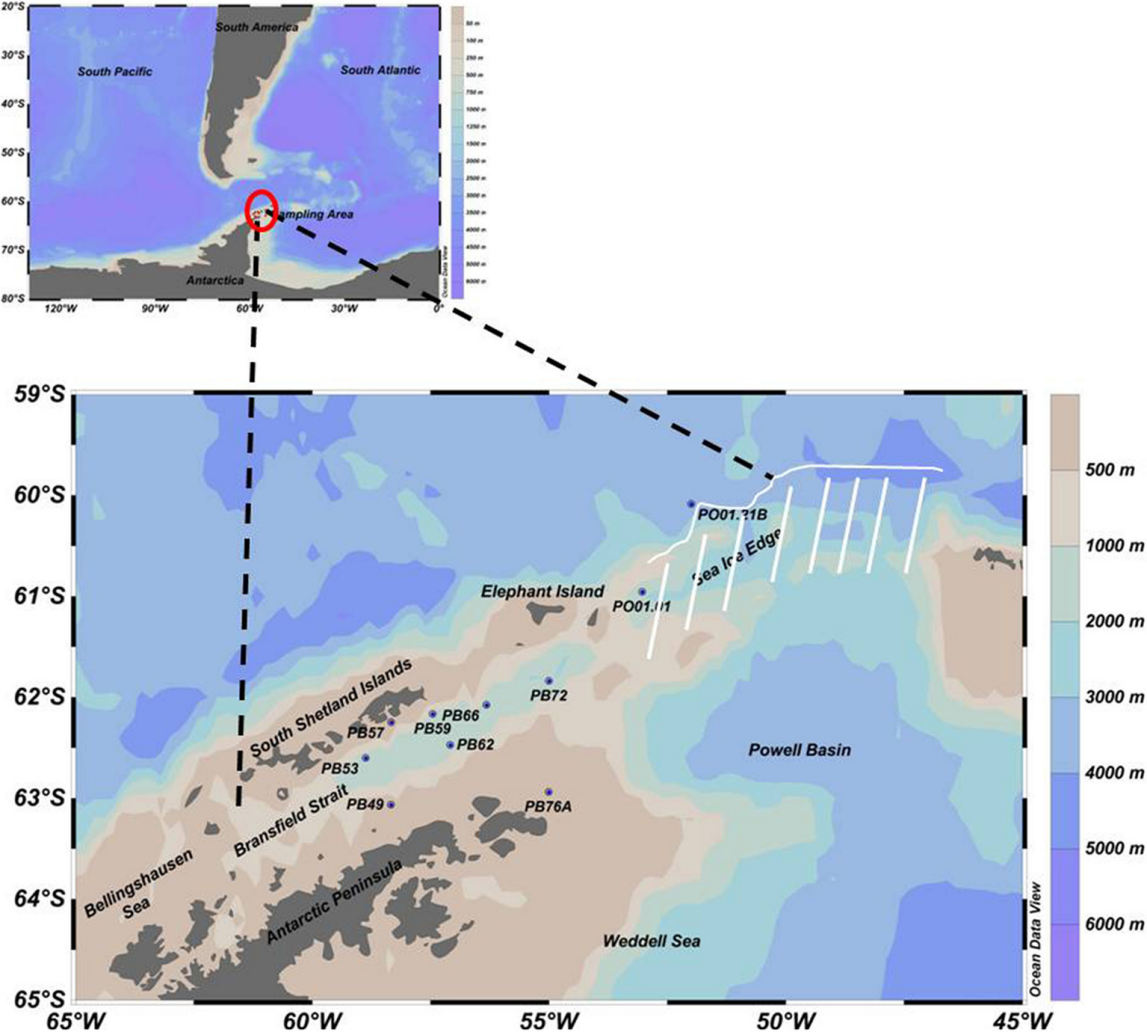
**Sampling map with 10 selected oceanographic stations in the Southern Ocean, from the Bransfield Strait (northwest of the AP) to the open ocean near the sea ice edge (north of the AP), represented by the white line**. Due to ice, navigation was impossible in the region indicated by the white stripes.

The research cruise was conducted by the Brazilian Navy vessel Npo. Almirante Maximiano (H44) during the austral summer, February to March 2013. Ten oceanographic stations were selected (PB49, PB53, PB57, PB59, PB62, PB66, PB72, PB76A, PO01.01, PO01.21B—see Figure 1) from the Bransfield Strait (located in the northwest of the Antarctic Peninsula) to the open ocean and near the sea ice edge (north of the AP), to be able to evaluate changes in the microbial community along an approximately 500 km long transect. Seawater and physical data (temperature, salinity and dissolved oxygen) were collected using a combined Sea-Bird CTD/Carrousel 911 system equipped with 24 5-l Niskin bottles. Sampling depths (surface, maximum and minimum of dissolved oxygen and near the bottom) were selected according to the CTD profiles. For microbial diversity approximately 1.8 l of seawater were filtered onto Sterivex filters (Millipore) with a pore size of 0.2 μm using a peristaltic pump. Filters were immediately frozen at −80°C. Due to competing water demands on the cruise, the data are from single, unreplicated samples. In general, within-sample replication for water samples in this area yields to low variability in the community composition (Luria et al., 2014).

### Environmental factors

Concentrations of inorganic nutrients (nitrate, nitrite, ammonia, silicate, and phosphate) were determined using a flow injection autoanalyzer (FIAstar 5000, Foss Tecator, Denmark). Samples for chlorophyll-a were filtered onto Whatman GF/F filters under vacuum pressure and analyzed using a Shimadzu High Performance Liquid Chromatography (HPLC).

### DNA extraction and sequencing

For DNA extraction, a protocol previously developed by Boström et al. (2004) and Manganelli et al. (2009) for low biomass samples was slightly modified by replacing t-RNA with linear acrylamide as co-precipitant. The DNA concentration in each sample was quantified by a fluorescent PicoGreen assay (Invitrogen), using a thermocycler (MX3005P, Stratagene). The composition of the microbial community was analyzed using a 16S rRNA gene based approach. Amplicons for sequencing were generated using the universal primers S-D-Bact-0785-a-S-18 and S-*-Univ-1392-a-A-15, recently identified as a suitable primer pair for Bacteria and Archaea after a thorough evaluation (Klindworth et al., 2013). This primer pair amplifies a 608 bp product and targets V5-V8 hypervariable regions of bacterial and archaeal 16S rRNA, with overall coverages of 86.5% and 67.0%, respectively (SILVA TestPrime 1.0, http://www.arb-silva.de/search/testprime/, tested in October 2014, allowing for 1 mismatch with no mismatches in the last 4 bases of the 3′end). A single-step 28 cycles PCR using HotStarTaq Plus Master Mix Kit (Qiagen, Valencia, CA) was used on 5 ng of DNA under the following conditions: 94°C for 3 min, followed by 28 cycles of 94°C for 30 s; 53°C for 40 s and 72°C for 1 min; after which a final elongation step at 72°C for 5 min was performed. Following PCR, all barcoded amplicon products (see Supplement Table 1 for details) from different samples were quantified using Qubit (Life Technologies), mixed in equal concentrations and purified using Agencourt Ampure beads (Agencourt Bioscience Corporation, MA, USA). After these steps, samples were sequenced using FLX titanium instruments and reagents (Roche 454), following the manufacturer's guidelines. The PCR and sequencing were carried out at Molecular Research Lab (Shallowater, Texas, USA). All sequence data have been deposited in the National Center for Biotechnology Information Sequence Read Archives (SRA) under BioProject ID PRJNA269510.

### 16S rRNA gene reads processing and statistical analyses

The 16S rRNA gene sequences were denoised with Acacia using the default parameters (Bragg et al., 2012) and further processed in the QIIME 1.7.0 pipeline (Caporaso et al., 2010). Reads were filtered for length (400 ≤ length ≤ 1000), quality score (mean, ≥25), number of ambiguous bases (=0), and length of homopolymer runs (<6). Sequences were clustered at 97% similarity using usearch (Edgar, 2010) including *de novo* and reference-based chimera checking. Taxonomy was assigned to each Operational Taxonomic Unit (OTU) using RDP classifier (Wang et al., 2007) with the Greengenes 13_5 database (McDonald et al., 2012). Sequences assigned to chloroplasts and mitochondria were removed from the dataset for further analysis. Multiple rarefactions at 594 reads per sample were conducted for alpha- and beta-diversity analyses. Differences in alpha-diversity estimates between groups of samples were tested using Student t-test in R (version 3.0.1). The weighted normalized Unifrac distance (Lozupone and Knight, 2005) was used for UPGMA (Unweighted Pair Group Method with Arithmetic Mean) cluster analysis with 100 resamplings and adonis statistical test with 999 permutations. The program iTOL (Interactive Tree Of Life, version 2.1, (available at http://itol.embl.de/index.shtml) was used for dendrogram visualization (Letunic and Bork, 2007, 2011). In addition, we plotted the weighted normalized UniFrac distances of all surface samples, which were collected at the same water depth (5 m), against geographical distance. Geographical distance is the actual distance between two stations, and it was calculated from latitude and longitude, assuming a spherical Earth, by using the following formula: d = acos(sin φ 1 · sin φ 2 + cos φ 1 · cos φ 2 · cos Δ λ) · R, where φ 1 is latitude of station 1, φ 2 is latitude of station 2, Δ λ is the difference in longitude, and R is the radius of the Earth. Non-metric multidimensional scaling (nMDS) was performed in R (version 3.0.1) on a Bray-Curtis dissimilarity matrix, with fitting of the environmental parameters using the envfit function from the vegan package (Oksanen et al., 2013). Differences in the relative abundances of taxa between groups were evaluated with a Welch's *t*-test as implemented in the STAMP program (Parks and Beiko, 2010), with FDR correction of the *p*-values to account for multiple testing, and filtering of the results based on effect sizes (difference between proportions >1 or ratio of proportions >4).

## Results

### Sampling site characteristics: environmental factors

**Figure 2:**
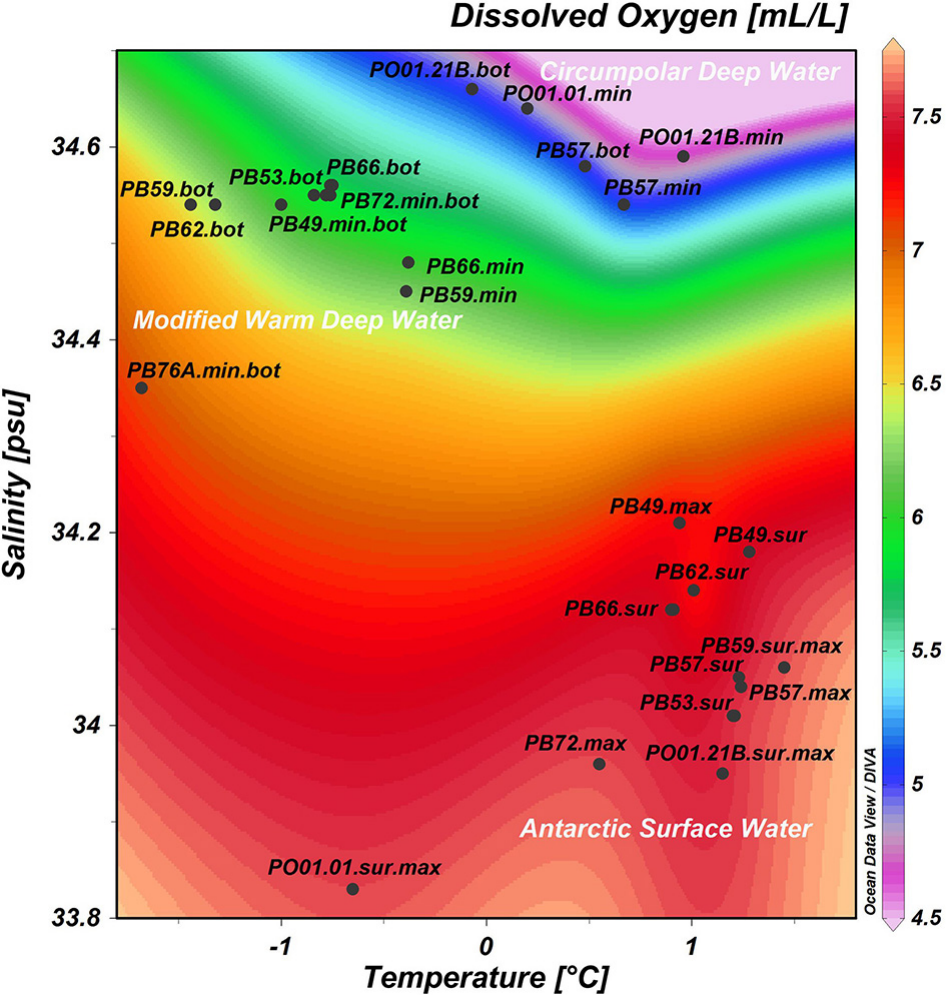
**Samples distribution according to *in situ* measurements of temperature (°C), salinity (psu) and dissolved oxygen (mL.L^−1^)**. In white: water masses sampled in the Southern Ocean. Sur, surface; max, maximum of dissolved oxygen; min, minimum of dissolved oxygen; bot, near the bottom.

The surface waters in Bransfield Strait were characterized by elevated temperatures and higher concentrations of oxygen, nitrite, ammonia and phytoplankton biomass (chlorophyll-a), when compared to the deep waters, which exhibited higher salinity as well as higher concentrations of nitrate, phosphate and silicate (Table 1). The highest chlorophyll-a value (5.75 mg.m^−3^) was found at the oceanographic station closest to the continent (PB57, at the Admiralty Bay entrance—South Shetland Islands). We found the highest concentration of silicate (66.36 μM, station PO01.21B) in the deepest sample (3012 m) close to the sea ice edge north of the AP (Table 1). When all the 30 samples were plotted according to *in situ* temperature, salinity, and dissolved oxygen, three groups of samples could clearly be separated, indicating that they belong to different water masses (Figure 2). The first group was influenced by the Antarctic Surface Water (AASW), with temperatures lower than 1°C, salinities between 33.0 and 34.5 psu, and a water depth greater than 200 m (Gordon and Huber, 1995). The second group of samples was affected by the Modified Deep Warm Water (mWDW), transitional waters with temperatures from −0.7 to −1.7°C and salinities between 34.4 and 34.6 psu (Robertson et al., 2002; Duarte, 2006). The third group was influenced by the Circumpolar Deep Water (CDW), with temperatures of 0.2–1.7°C and salinities of 34.5–34.8 psu (Santoso et al., 2006).

**Table 1:** **Environmental parameters measured for each sample in the Western Antarctic Peninsula (WAP)**.

**Station**	**Latitude (°N)**	**Longitude (°E)**	**Depth (m)**	**Corresponding depth**	**Temperature (°C)**	**Salinity (psu)**	**Dissolved Oxygen (mL.L^−1^)**	**Ammonia (μM)**	**Nitrite (μM)**	**Nitrate (μM)**	**Phosphate (μM)**	**Silicate (μM)**	**Chl-a (mg.m^−**3**^)**
PB49	−63.0651	−58.3329	5	Sur	1.28	34.18	7.44	0.53	0.28	20.42	2.26	33.59	0.90
PB49	−63.0651	−58.3329	30	Max	0.94	34.21	7.37	0.48	0.21	20.98	1.99	36.08	0.50
PB49	−63.0651	−58.3329	720	Min.bot	−1.00	34.54	5.99	0.00	0.09	27.18	2.41	41.70	0.00
PB53	−62.6014	−58.8678	5	Sur	1.21	34.01	7.57	0.43	0.13	20.83	1.32	33.00	1.41
PB53	−62.6014	−58.8678	34	Max	1.20	34.01	7.55	0.39	0.10	19.99	1.32	33.01	0.50
PB53	−62.6014	−58.8678	613	Min	−0.76	34.56	5.81	0.12	0.00	27.65	2.01	42.75	0.00
PB53	−62.6014	−58.8678	800	Bot	−0.84	34.55	5.83	0.00	0.00	27.61	2.14	44.76	0.00
PB57	−62.2477	−58.3272	5	Sur	1.23	34.05	7.51	0.33	0.32	23.41	1.59	32.01	5.75
PB57	−62.2477	−58.3272	10	Max	1.24	34.04	7.51	0.45	0.25	24.21	1.72	32.02	2.73
PB57	−62.2477	−58.3272	330	Min	0.67	34.54	5.00	0.11	0.07	32.76	2.32	49.01	0.00
PB57	−62.2477	−58.3272	553	Bot	0.48	34.58	5.06	0.11	0.06	32.49	2.76	47.11	0.00
PB59	−62.1658	−57.4501	5	Sur.max	1.45	34.06	7.58	0.52	0.14	22.91	1.33	31.27	2.48
PB59	−62.1658	−57.4501	225	Min	−0.39	34.45	6.30	0.18	0.11	29.87	1.85	38.49	0.00
PB59	−62.1658	−57.4501	1848	Bot	−1.44	34.54	6.44	0.00	0.00	30.66	2.43	39.14	0.00
PB62	−62.4734	−57.0826	5	Sur.max	1.01	34.14	7.00	0.70	0.18	24.08	1.79	36.41	1.11
PB62	−62.4734	−57.0826	700	Min	−0.78	34.55	5.85	0.00	0.00	31.19	2.41	43.06	0.00
PB62	−62.4734	−57.0826	947	Bot	−1.32	34.54	6.35	0.00	0.00	31.11	2.10	41.82	0.00
PB66	−62.0756	−56.318	6	Sur	0.90	34.12	7.40	0.81	0.33	25.33	1.99	38.74	1.14
PB66	−62.0756	−56.318	35	Max	0.91	34.12	7.39	0.60	0.18	24.15	2.99	33.99	0.30
PB66	−62.0756	−56.318	250	Min	−0.38	34.48	5.94	0.00	0.01	30.80	2.28	43.91	0.00
PB66	−62.0756	−56.318	2129	Bot	−0.75	34.56	5.80	0.00	0.00	31.30	2.35	47.14	0.00
PB72	−61.8382	−54.9961	5	Sur	0.55	33.96	7.58	1.18	0.25	23.73	1.59	32.41	1.15
PB72	−61.8382	−54.9961	15	Max	0.55	33.96	7.58	0.83	0.19	24.65	1.61	36.52	1.30
PB72	−61.8382	−54.9961	2188	Min.bot	−0.76	34.55	5.82	0.00	0.00	31.86	2.05	41.16	0.00
PB76A	−62.9403	−55.0019	208	Min.bot	−1.68	34.35	7.06	0.32	0.11	30.33	2.46	38.65	0.00
PO01.01	−60.9575	−53.0289	5	Sur.max	−0.65	33.83	7.57	1.18	0.15	26.10	1.78	34.89	1.15
PO01.01	−60.9575	−53.0289	828	Min	0.20	34.64	4.90	0.13	0.00	30.80	2.13	47.63	0.00
PO01.21B	−60.0863	−51.994	5	Sur.max	1.15	33.95	7.48	0.33	0.13	24.93	1.87	33.15	0.97
PO01.21B	−60.0863	−51.994	300	Min	0.96	34.59	4.68	0.10	0.09	32.49	2.52	38.43	0.00
PO01.21B	−60.0863	−51.994	3012	Bot	−0.07	34.66	5.02	0.00	0.00	33.23	2.56	66.36	0.00

Latitude (°), Longitude (°), Depth (m), Corresponding depth, Temperature (°C), Salinity (psu), Dissolved Oxygen (mL.L^−1^), Ammonia (μM), Nitrite (μM), Nitrate (μM), Phosphate (μM), silicate (μM), and Chl-a, chlorophyll-a (mg.m^−3^). Sur, surface; max, maximum of dissolved oxygen; min, minimum of dissolved oxygen; bot, near the bottom.

### Overall diversity

**Figure 3:**
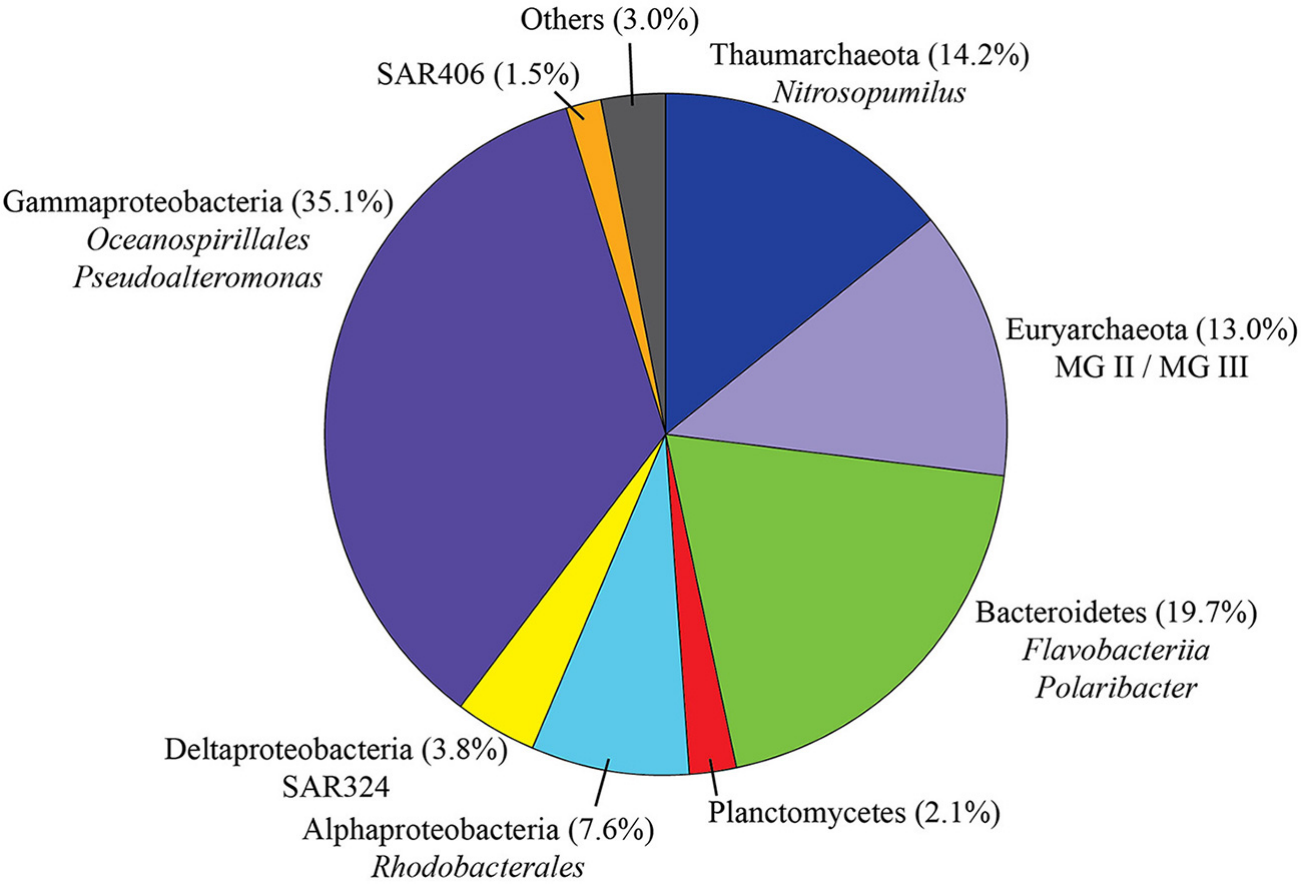
**Taxonomic distribution of all sequences retrieved during this study (*n* = 146,287)**. “Others” comprises taxa accounting for less than 1% of the total.

After quality checking and data filtering, a total of 146,287 sequences were obtained from 30 water samples, with an average of 4876 reads per sample (range 594–10,574 reads per sample). Clustering of these reads at 97% similarity threshold resulted in 1,506 OTUs: 172 for Archaea (11.4%) and 1,328 for Bacteria (88.2%). Considering all sequences retrieved in the present study (Figure 3), Gammaproteobacteria accounted for the largest fraction (35.1% of the total)—mainly represented by the orders Oceanospirillales (17.4%), Vibrionales (9.1%, dominated by the genus *Pseudoalteromonas)* and Alteromonadales (7.1%). Bacteroidetes was the second most represented class (19.7%), with representatives mostly from the order Flavobacteriales (19.2%, dominated by the psychrophilic and proteorhodopsin-containing *Polaribacter*). In addition, we also identified Alphaproteobacteria (7.6%, mainly represented by the order Rhodobacterales and to a lesser extent SAR11), Deltaproteobacteria (3.8%, SAR324), Planctomycetes (2.1%), SAR 406 (1.5%), and other taxa with contributions lower than 1%. Archaea were mainly represented by Thaumarchaeota (*Nitrosopumilus*) with 14.2% and Euryarchaeota (class Thermoplasmata, Marine Groups II and III) with 13% of the total number of sequences.

### Alpha diversity

After rarefaction to even sequencing depth, the number of observed OTUs per sample ranged from 63 to 167 (Table 2). Richness and diversity were significantly greater in samples originating from deep waters (oxygen minimum and seafloor) than in samples collected at shallower depth (surface and oxygen maximum). Considering all the indices, the lowest values of diversity were found at the following oceanographic stations: PB53.Sur, PB57.Sur, PB59.Sur.Max, PB72.Sur, PO01.01.Sur.Max, and PO.21B.Sur.Max, which were influenced by the Antarctic Surface Water (AASW), i.e., characterized by higher temperatures and dissolved oxygen and lower salinities. The highest values were found at PB49.Min.Bot, PB53.Bot, and PB62.Bot, influenced by the modified Warm Deep Water, with relatively lower temperatures and oxygen and higher salinities (Figure 2).

**Table 2:** **Values of alpha-diversity estimates**.

**Sample ID**	**Depth (m)**	**OTUs**	**Chao1**	**Shannon**	**Simpson**
PB76A.min.bot	208	107.70^a^	204.02^a^	5.14^a^	0.94^a^
PB49.sur	5	69.50	110.07	3.99	0.87
PB49.max	30	71.30	122.96	3.96	0.83
PB49.min.bot	720	149.90	261.03	5.90	0.96
PB53.sur	5	63.40	85.05	4.00	0.87
PB53.max	34	70.70	115.84	4.08	0.85
PB53.min	613	155.40	292.60	5.99	0.97
PB53.bot	800	167.80	288.59	6.17	0.97
PB57.sur	5	74.80	116.30	4.64	0.92
PB57.max	10	95.70	164.51	4.88	0.92
PB57.min	330	129.10	238.80	5.47	0.94
PB57.bot	553	127.10	231.39	5.57	0.95
PB59.sur.max	5	69.70	92.46	4.73	0.94
PB59.min	225	113.50	197.04	5.00	0.91
PB59.bot	1848	135.10	232.67	5.57	0.94
PB62.sur.max	5	82.00	122.95	4.72	0.92
PB62.min	700	140.20	244.93	5.82	0.96
PB62.bot	947	158.60	269.59	6.11	0.96
PB66.sur	6	82.20	123.46	4.75	0.92
PB66.max	35	77.10	114.77	4.57	0.91
PB66.min	250	113.90	191.64	5.07	0.91
PB66.bot	2129	147.30	247.99	5.83	0.95
PB72.sur	5	68.90	104.68	3.89	0.81
PB72.max	15	81.70	128.82	4.58	0.90
PB72.min.bot	2188	146.80	278.32	5.67	0.95
PO01.01.sur.max	5	64.10	106.51	3.84	0.81
PO01.01.min	828	118.30	189.87	5.47	0.95
PO01.21B.sur.max	5	71.20	85.13	4.93	0.94
PO01.21B.min	300	103.40	165.85	5.24	0.94
PO01.21B.bot	3012	135.70	257.28	5.52	0.94
***T*-TEST^b^**					
Average “deep”		134.36	236.98	5.60	0.95
Average “shallow”		74.75	113.82	4.40	0.89
t-statistics		11.18	11.24	8.47	4.57
*P*-value		<0.0001	<0.0001	<0.0001	0.0003

Sur, surface; max, maximum of dissolved oxygen; min, minimum of dissolved oxygen; bot, near the bottom.

a All values are averages based on 10 rarefactions at 594 sequences per sample.

b Results of Student t-test, testing the null hypothesis of equality of means in “deep” samples (oxygen minimum or near the bottom) vs. “shallow” samples (surface or oxygen maximum).

### Microbial community structure and environmental parameters

**Figure 4:**
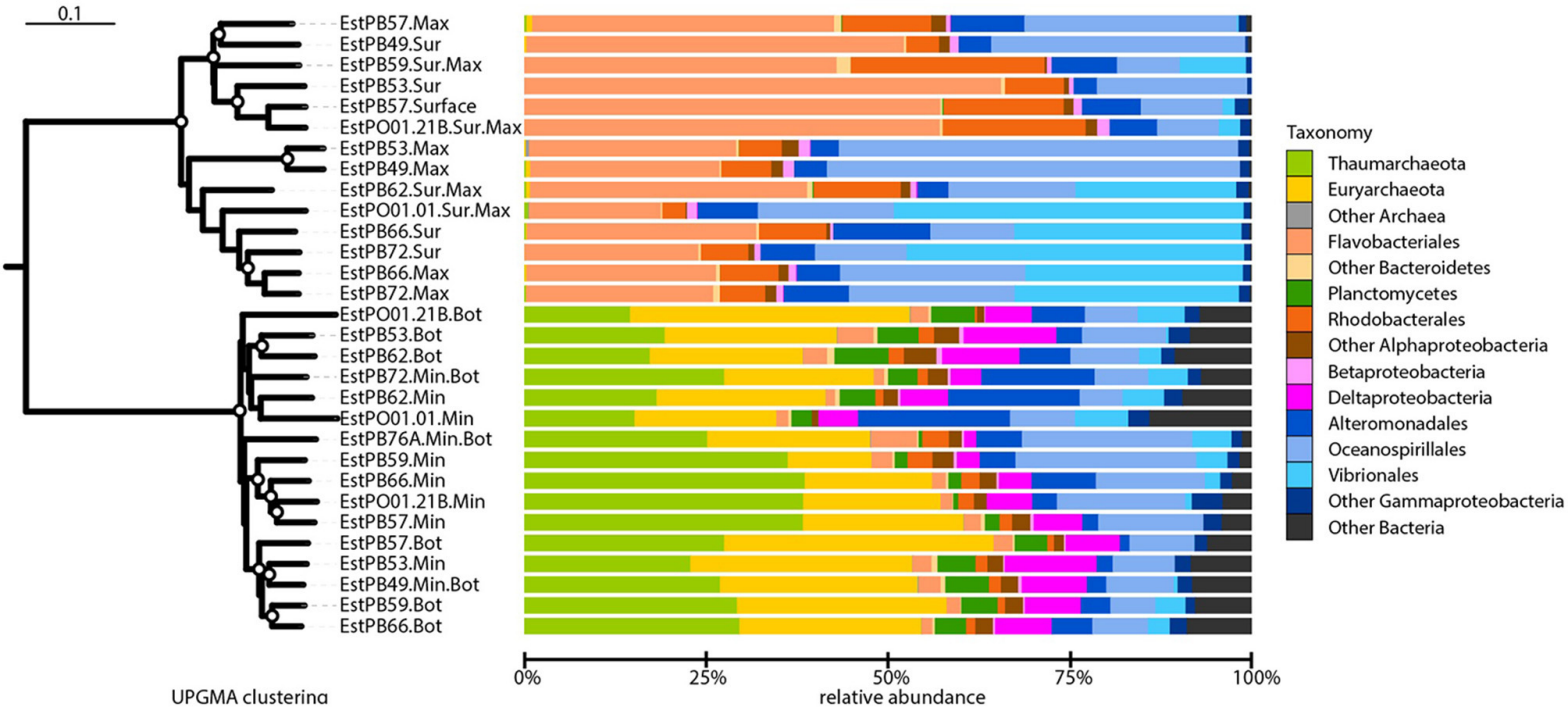
**Beta-diversity analysis and taxonomic composition of Southern Ocean samples collected from near the seafloor (Bot), at the minimum or maximum oxygen depth (Min or Max, respectively), or at the surface (Sur)**. Samples IDs are as shown in Table 2. **Left**: unweighted pair group method with arithmetic mean (UPGMA) dendrogram based on weighted-normalized unifrac distances. Bootstrap values higher than 50% based on 100 resamplings are shown as white circles on the branches. The bar represents 10% dissimilarity. **Right**: relative abundances of archaeal and bacterial taxa. Some major taxa are shown at the order level for better taxonomic resolution.

**Figure 5:**
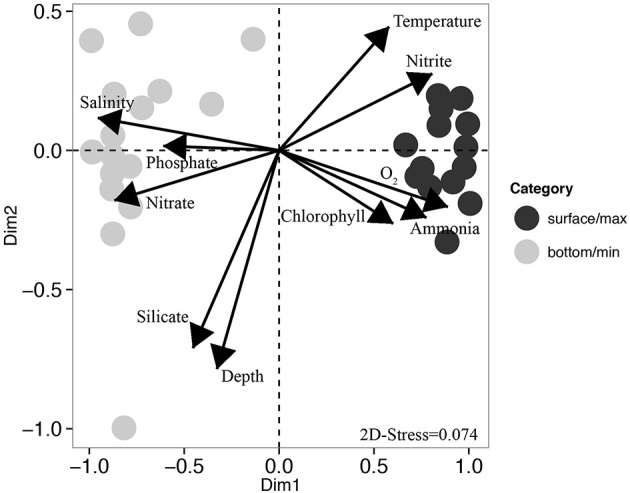
**Non-metric multidimensional scaling (nMDS) ordination**. Dark circles represent samples collected either at the surface or at the oxygen maximum, and gray circles represent samples collected either at the oxygen minimum or close to the seafloor. Each arrow represents one environmental gradient significantly correlated to the ordination (envfit, *p* < 0.05). The arrow points to the direction of the most rapid change in the environment (direction of the gradient) and its length is proportional to the correlation between ordination and environmental variable (strength of the gradient).

Beta-diversity analysis using UPGMA clustering revealed a clear separation of samples originating either from the surface or oxygen maximum from samples originating either from near the seafloor or the oxygen minimum (Figure 4), which was statistically supported by adonis analysis (*F* = 28.76, *r*^2^ = 0.51, *P* < 0.001). This was further corroborated by the non-metric multidimensional scaling (nMDS) analyses, which also revealed a clear distinction between surface and deeper samples (Figure 5). nMDS further revealed that all the environmental parameters were significantly (*p* < 0.05) correlated with microbial community composition, although the highest correlations were observed for salinity (*r*^2^ = 0.93), dissolved oxygen (*r*^2^ = 0.83), and nitrate (*r*^2^ = 0.79). The surface samples were dominated by Gammaproteobacteria (relative abundance between 19.3 and 76.0%, Figure 4), Bacteroidetes (18.3–66.1%), and Alphaproteobacteria (3.4–27.0%). Nearly 50% of the sequences from deeper samples belonged to archaeal OTUs, with relative abundance ranging from 14.6 to 38.6% for Thaumarchaeota and from 11.5 to 38.5% for Euryarchaeota. Deltaproteobacteria and Planctomycetes were also mainly found in deep waters. As in surface waters, the class Gammaproteobacteria was also found to be abundant in the deep waters, with percentages varying from 12.0 to 40.1%. For surface samples (all collected at 5 m water depth), weighted normalized Unifrac distance between pairs of samples was plotted against geographical distance (Supplementary Figure 1). In general, Unifrac distance was positively correlated to geographical distance, except for 5 outlier pairs of samples (in red in Supplementary Figure 1A). These five outlier pairs were between PO01.21B Sur.Max and PB62 Sur.Max, PB59 Sur.Max, PB57 Surface, PB49 Surface, and PB53 Surface. When they were removed from the analysis, there was a strong correlation between Unifrac distance and geographical distance (Supplementary Figure 1B).

**Figure 6:**
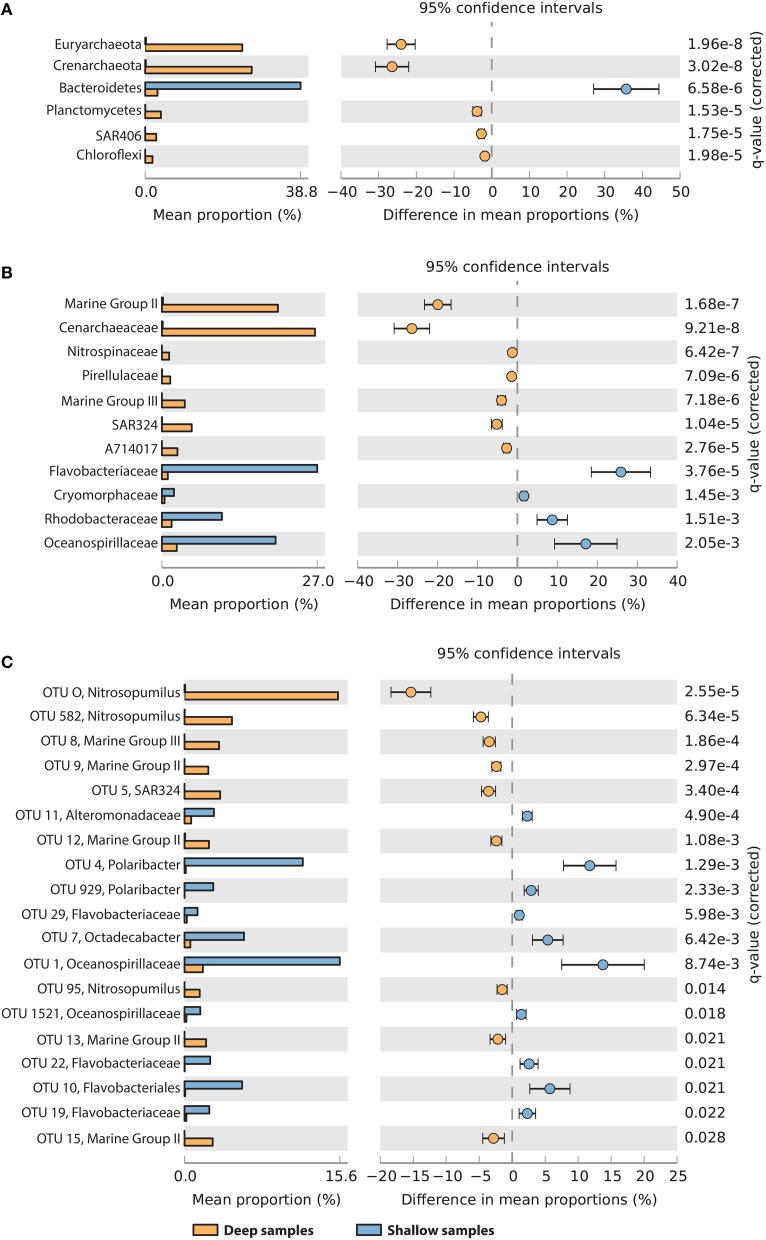
**Taxa showing significantly different relative abundances between “deep” samples (yellow, oxygen minimum, or seafloor) and “shallow” samples (blue, oxygen maximum or surface), at the phylum (A), family (B), and OTU (C) levels**. For OTUs, the best taxonomic assignment is reported. Data were compared using Welch's *t*-test with FDR correction of *p*-values. Differences were considered significant if *q* < 0.05 and filtered for effect sizes (difference between proportions >1 or ratio between proportions >4).

To determine which taxa were most contributing to the observed difference between shallow (surface or maximum oxygen) and deep samples (seafloor or minimum oxygen), we tested for difference in the relative abundances between the two groups, at the phylum, family and OTU taxonomic resolution (Figure 6). At the phylum and family levels (Figures 6A,B), deep samples were significantly enriched in Euryarchaeota (Marine Group II and III), Crenarchaeota (Cenarchaeacea), Planctomycetes (Pirellulaceae), SAR406 (group AT714017), and Chloroflexi. Shallow samples showed higher contributions of Bacteroidetes (Flavobacteriaceae and Cryomorphaceae), Rhodobacteraceae and Oceanospirillaceae (Figures 6A,B). In total, 19 OTUs showed significant variations between deep and shallow samples (Figure 6C). Nine of them were more abundant in deep waters than in shallow waters, including 8 archaeal OTUs (related to *Nitrosopumilus*, MG II and MG III) and one deltaproteobacterial OTU (related to SAR324). The remaining 10 OTUs were significantly more abundant in shallow samples, including 6 flavobacterial OTUs (2 related to *Polaribacter*), 3 gammaproteobacterial OTUs (related to Oceanospirillales and Alteromonadales) and one alphaproteobacterial OTU (*Octadecabacter*).

## Discussion

### Surface vs. deep-sea microbial community diversity and structure

The present work showed a clear distinction of the microbial community structure according to depth and characteristics of the water masses. The clear differences in microbial community structure based on depth were consistent with previous studies of strong vertical stratification in community composition (DeLong et al., 2006; Brown et al., 2009; Luria et al., 2014). Results from nMDS analyses showed that the differences in microbial communities could be explained by a combination of environmental factors, with salinity, dissolved oxygen, and nitrate—all factors that are influenced by water column vertical stratification—having the strongest influence. A similar trend was observed by Luria et al. (2014), who found that salinity and silicate were the factors best explaining the differences in bacterial community composition, whereas for the eukaryotic community composition it was temperature and oxygen. However, while some of the factors determined in our study might directly influence the community composition, for example the oxygen concentration, we cannot exclude the possibility that they might co-vary with other parameters that we did not measure, like the organic matter composition.

Communities from above 100 m depth were dominated by Gammaproteobacteria (mainly from the orders Oceanospirillales, Vibrionales and Alteromonadales), Bacteroidetes (mainly *Polaribacter*-related) and Alphaproteobacteria, whereas Archaea and Gammaproteobacteria prevailed in deeper waters, confirming results of previous studies (Ghiglione and Murray, 2012; Grzymski et al., 2012; Jamieson et al., 2012; Williams et al., 2012; Wilkins et al., 2013b; Luria et al., 2014). The identified microbes in the surface waters correspond to a predominantly heterotrophic summer community subsisting on organic matter produced by photoautotrophs, a model previously derived for a coastal site off Palmer Station using “omic” approaches (Grzymski et al., 2012; Williams et al., 2012). In contrast, the deep-water communities correspond to the winter surface community, which is characterized by a higher proportion of potentially chemolithoautotrophic bacteria and archaea (Grzymski et al., 2012; Williams et al., 2012). In fact, the pronounced differences in composition when comparing the austral summer and winter microbial communities and the similarities between the temporal (seasonal) and the spatial (depth) variation has been noted early on by Murray et al. (1998), and been confirmed in a number of subsequent studies (Murray and Grzymski, 2007; Manganelli et al., 2009; Ghiglione and Murray, 2012; Grzymski et al., 2012; Williams et al., 2012). A similar pattern was recently reported by Luria et al. (2014), who sampled at the surface and at 100 m just below the euphotic zone at 4 stations on the continental shelf of the WAP. Luria et al. (2014) speculate that the reason the winter surface community resembles the deeper communities is due to the fact that the summer surface community develops in the cold, salty Winter Water as it warms and freshens in spring and summer. However, in contrast to the deep communities identified in the present study, the summer communities at 100 m reported by Luria et al. (2014) still contain a signal of the community in the winter surface layer, which becomes isolated at about 100 m depth by the summer warming, for example in the form of a larger contribution by Alphaproteobacteria and Bacteroidetes, indicating that the base of the photic zone represents a transition between the summer surface community and the deep-water communities. The fact that the communities in the deep-ocean are similar to the winter surface community further suggests that the conditions in the water column below the photic zone are quite homogenous, and it appears that deep-water communities are carried to the surface, where they can thrive in the winter and get outcompeted by the summer community (Murray et al., 1998; Luria et al., 2014).

### Surface community

Communities found in surface and maximum oxygen samples were dominated by heterotrophic bacteria, likely consuming the phytoplankton-derived organic matter. This includes members of the Oceanospirillales and Alteromonadales within Gammaproteobacteria, which were significantly more abundant in shallow waters than in deep samples. Gammaproteobacteria are known to play an important ecological role in the degradation of organic carbon by producing extracellular hydrolytic enzymes (such as amylases, proteases, lipases, and DNAses) (Dang et al., 2009), although some Oceanospirillales have also been shown to possess genes for carbon fixation (Calvin cycle), and thus have the potential to grow as chemoautotrophs (Swan et al., 2011). The greater relative abundance of Bacteroidetes at the surface compared to deeper waters also likely reflects their potential association with phytoplankton, since many cultivated species of this phylum have specialized in the degradation of high molecular weight compounds derived from primary producers (Wilkins et al., 2013b). The most representative genus belonging to the Bacteroidetes was found to be *Polaribacter*—a gas-vacuolated, proteorhodopsin-containing Flavobacteria that is prevalent in Antarctica and Arctic seawater (Wilkins et al., 2013b). Grzymski et al. (2012) reported sequences affiliated with the genus *Polaribacter* only in summer (comprising 11% of the 16S rRNA gene reads), and speculate that they are seeded from phytoplankton blooms following the melting of sea ice. The class Alphaproteobacteria was mainly represented by the order Rhodobacterales, known as a common and dominant primary surface colonizing group (Dang et al., 2008). We also identified sequences belonging to the SAR11 clade, which has been identified as a major player of microbial communities in the surface ocean, including the Southern Ocean (Brown et al., 2012).

Interestingly, we found that the surface communities became more dissimilar from each other with geographical distance, indicating that different regions harbor distinct communities. This is in contrast to results reported by Luria et al. (2014), who did not find a relationship with geographical distance. Luria et al. (2014) argue that similar conditions observed in summer lead to similar surface communities, whereas differences in communities might be expected at times when conditions change, for example in response to transitions between seasons (spring-summer or summer-fall). We sampled in late summer at the transition to fall, which would fit with this scenario. As an exception to the trend with geographical distance, we found that the surface community of PO01.21B, which was the station furthest away from shore, was more similar to samples obtained closer to shore than distance would suggest. One possible explanation could be that PO01.21B was located 30 m away from the sea ice edge, leading to the development of communities that are more similar to communities in coastal waters, which appear to be dominated by Flavobacteria. Also, these stations are all characterized by elevated temperature and chlorophyll-a concentrations, suggesting that increased activity due to higher temperatures and the availability of organic matter might be driving this difference.

### Deep community

The deep-water masses we sampled (Circumpolar Deep Water, Antarctic Intermediate Water, and Antarctic Bottom Water) were dominated by Thaumarchaeota (*Nitrosopumilus*), Euryarchaeota (MGII and III), and Gammaproteobacteria, largely in line with the few studies that have analyzed the microbial communities of deep waters of the Southern Ocean (López-García et al., 2001; Manganelli et al., 2009). Thaumarchaeota may be responsible for nitrification in the deep ocean (Könneke et al., 2005; Wuchter et al., 2006; Nakagawa et al., 2007; Kalanetra et al., 2009), whereas Euryarchaeota belonging to the MGII clade have been shown to be motile, proteorhodopsin-containing photoheterotrophs that have the potential to degrade proteins and lipids (Iverson et al., 2012). In contrast to deep waters, we detected almost no 16S rRNA genes from Archaea above 100 m depth, in line with previous studies sampling surfaces communities in austral summer (Murray et al., 1998; Murray and Grzymski, 2007; Manganelli et al., 2009; Ghiglione and Murray, 2012; Grzymski et al., 2012; Williams et al., 2012). The ammonia-oxidizing Thaumarchaeota have been shown to be very sensitive to photoinhibition (Merbt et al., 2012), which could explain their decline during periods of extended illumination, for example during the summer in Southern Ocean waters when light levels are highest (Murray et al., 1998; Grzymski et al., 2012). In line with this hypothesis, metaproteomic analysis found that Thaumarchaeota/*Nitrosopumilus maritimus* represented 30% of all microbial proteins in winter coastal Antarctic Peninsula samples, whereas no proteins were detected in the summer (Williams et al., 2012). On the other hand, the fact that we did not find any euryarchaeotal sequences in the surface samples is somewhat surprising, considering that some have been shown to contain proteorhodopsin, and thus could grow as photoheterotrophs (Iverson et al., 2012). Indeed, in contrast to Thaumarchaeota, Euryarchaeota have been frequently identified in surface waters in other oceanic regions, including the summer surface community in the Southern Drake Passage (Manganelli et al., 2009). This discrepancy might in part be explained by the partial coverage of the primers we used toward Euryarchaeota (61.9%). However, their complete absence in surface samples in our dataset suggests that further work is required to elucidate the factors that govern the distribution of Euryarchaeota in the Southern Ocean. Although less prevalent than Archaea and Gammaproteobacteria, Deltaproteobacteria (dominated by the SAR324 clade) and Planctomycetes were almost exclusively found in deep waters. Deltaproteobacterial sequences belonging to SAR324 have previously been found in deeper layers and also as part of the Antarctic surface winter community (López-García et al., 2001; Grzymski et al., 2012; Luria et al., 2014), where they are likely to contribute to chemosynthetic production due to their versatile metabolism (Swan et al., 2011; Sheik et al., 2014). We further detected sequences belonging to the SAR406 clade, which has been frequently identified in the deep-ocean (Rappé et al., 2000), including Antarctic deep waters (López-García et al., 2001). Their ecological role remains to be elucidated.

## Conclusions

We found a clear separation between surface and deeper samples of Bransfield Strait and near the ice edge north of the AP, considering the microbial composition, richness, and diversity. Communities originating from the transitional waters (from the modified Warm Deep Water) showed the highest diversity, whereas communities influenced by surface waters (Antarctic Surface Water) exhibited the lowest. A combination of environmental factors drives the microbial community structure in Bransfield Strait, especially salinity, dissolved oxygen, and nitrate which showed the highest correlation indices with the ordination. Among the groups identified in this study, Thaumarchaeota (*Nitrosopumilus*-related phylotypes), Gammaproteobacteria (Oceanospirillales), and Deltaproteobacteria (SAR 324) are likely to contribute to chemosynthetic production in deep waters of the Southern Ocean, and also at the surface in winter, when light is a limiting factor. On the other hand, groups like Bacteroidetes, Gammaproteobacteria (Vibrionales, Alteromodales), Euryarchaeota (MGII) and Alphaproteobacteria (Rhodobacterales) are likely to have a heterotrophic metabolism and contribute to organic matter degradation. Our results represent a baseline study of the microbial community in this critical area of the Southern Ocean, and also extend the recent survey of Luria et al. (2014) further to the north. These data will help to understand how the dynamic seascape of the Southern Ocean shapes the microbial community composition and provide an invaluable reference point to evaluate how the system might respond to future changes.

### Conflict of interest statement

The authors declare that the research was conducted in the absence of any commercial or financial relationships that could be construed as a potential conflict of interest.
