# Supplementary material for: Microbial diversity and community structure across environmental gradients in Bransfield Strait, Western Antarctic Peninsula
Source: Front Microbiol. 2014 Dec 16;5:647. doi: 10.3389/fmicb.2014.00647 (PMC4267279; doi:10.3389/fmicb.2014.00647)
Supplement: Supplementary file 1 [file Table1.DOCX]

**Supplementary Material**

**Table S1.** Barcode sequence and linker primer sequence for each sample.

| **Sample** | **Depth (m)** | **Barcode Sequence** | **Linker Primer Sequence** |
| --- | --- | --- | --- |
| PB76A.min.bot | 208 | GCGAGCTC | GGMTTAGATACCCBDGTA |
| PB49.sur | 5 | GCGATGCC | GGMTTAGATACCCBDGTA |
| PB49.max | 30 | GCGCACGC | GGMTTAGATACCCBDGTA |
| PB49.min.bot | 720 | GCGGAGAA | GGMTTAGATACCCBDGTA |
| PB53.sur | 5 | GCGGGCGA | GGMTTAGATACCCBDGTA |
| PB53.max | 34 | GCGGGTAT | GGMTTAGATACCCBDGTA |
| PB53.min | 613 | GCGGTGTC | GGMTTAGATACCCBDGTA |
| PB53.bot | 800 | GCGTGAAT | GGMTTAGATACCCBDGTA |
| PB57.sur | 5 | GCGTGAGT | GGMTTAGATACCCBDGTA |
| PB57.max | 10 | GCGTTGGA | GGMTTAGATACCCBDGTA |
| PB57.min | 330 | GCTAAGCT | GGMTTAGATACCCBDGTA |
| PB57.bot | 553 | GCTAGCTG | GGMTTAGATACCCBDGTA |
| PB59.sur.max | 5 | GCTCACAA | GGMTTAGATACCCBDGTA |
| PB59.min | 225 | GCTCACAC | GGMTTAGATACCCBDGTA |
| PB59.bot | 1848 | GCTCACTT | GGMTTAGATACCCBDGTA |
| PB62.sur.max | 5 | GCTGAAGC | GGMTTAGATACCCBDGTA |
| PB62.min | 700 | GCTGAGTA | GGMTTAGATACCCBDGTA |
| PB62.bot | 947 | GCTGCACA | GGMTTAGATACCCBDGTA |
| PB66.sur | 6 | GCTGCACC | GGMTTAGATACCCBDGTA |
| PB66.max | 35 | GCTGCCTC | GGMTTAGATACCCBDGTA |
| PB66.min | 250 | GCTGCTAA | GGMTTAGATACCCBDGTA |
| PB66.bot | 2129 | GCTGCTTC | GGMTTAGATACCCBDGTA |
| PB72.sur | 5 | GCTTATAA | GGMTTAGATACCCBDGTA |
| PB72.max | 15 | GCTTCAAT | GGMTTAGATACCCBDGTA |
| PB72.min.bot | 2188 | GCTTCAGA | GGMTTAGATACCCBDGTA |
| PO01.01.sur.max | 5 | GCTTCATT | GGMTTAGATACCCBDGTA |
| PO01.01.min | 828 | GCTTCGGT | GGMTTAGATACCCBDGTA |
| PO01.21B.sur.max | 5 | GGAATAGG | GGMTTAGATACCCBDGTA |
| PO01.21B.min | 300 | GGACAATC | GGMTTAGATACCCBDGTA |
| PO01.21B.bot | 3012 | GGACACGT | GGMTTAGATACCCBDGTA |
